# Celebrating 50 Years of Single-Channel Recording with the Patch Clamp

**DOI:** 10.1007/s00232-025-00362-3

**Published:** 2025-09-19

**Authors:** Luigi Catacuzzeno, Fabio Franciolini

**Affiliations:** Dipartimento di Chimica, Biologia e Biotecnologie, Universita’ di Perugia, Perugia, Italy

**Keywords:** Ion channels, Single-channel recording, Patch clamp, Channel kinetics, Markov models

## Abstract

Fifty years ago, Erwin Neher and Bert Sakmann published a *Nature* paper on their recording of discrete, step-like currents of a few picoamps passing through individual acetylcholine receptor channels of frog muscle fibers. This observation, the first on native channels, immediately ended the decade-long dispute about the presence of ion channels on cell membranes by convincing even the most reluctant scientists that this was indeed the case. More importantly, however, the ability to record single-channel currents revolutionized the study of ion channels because it enabled scientists to observe their behavior individually in real time. We could observe them change conformation, jumping from the closed state to the open state and back again. This level of detail provided an unprecedented understanding of the gating mechanisms, conductance, and kinetic properties of channels. This retrospective illustrates the scientific context in which all of this occurred as well as its immediate and current impact on the investigation of ion channels.

**Graphical Abstract:**

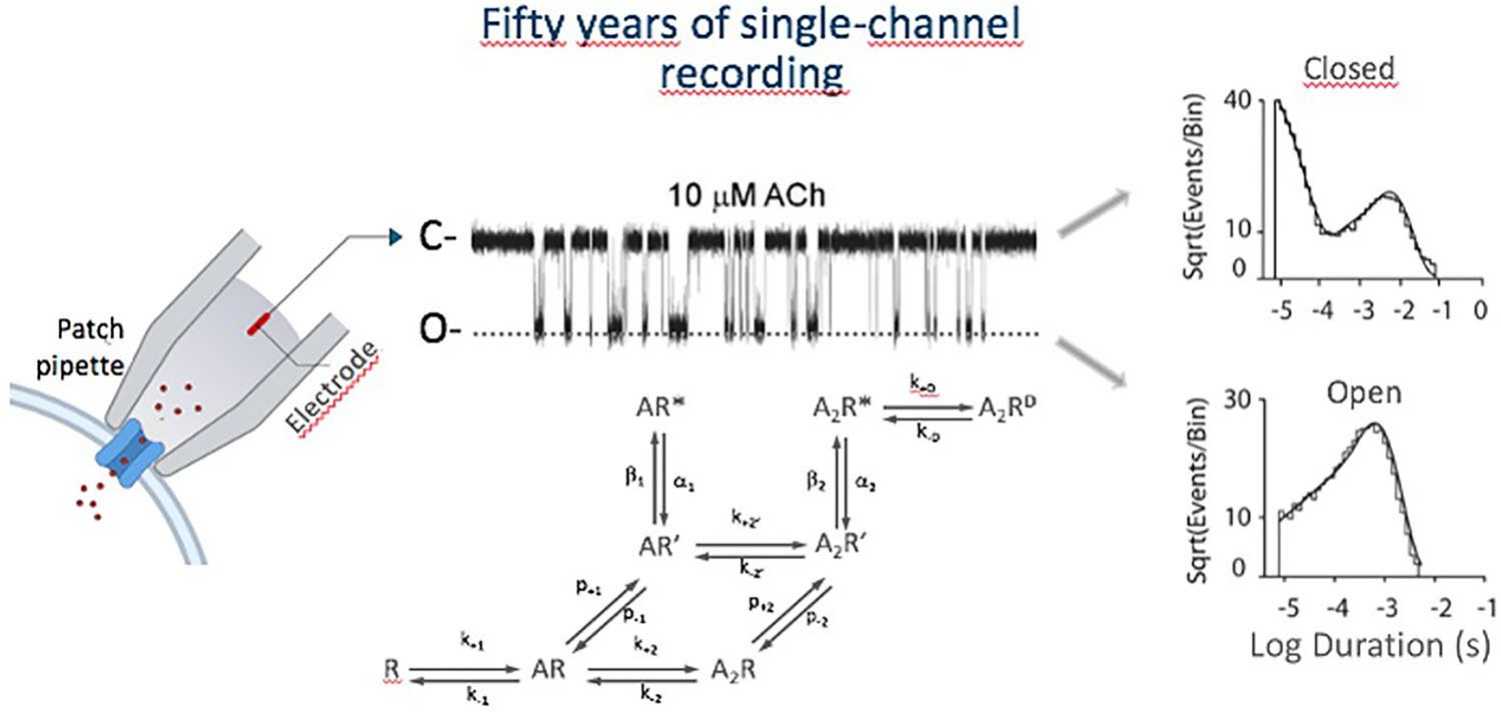

## Introduction

Recording elementary currents through single ion channels is one of the most eagerly pursued and rewarding goals in the field of membrane biophysics and cell physiology. To fully appreciate this achievement, we can look back to a time when ion channels did not exist for the scientific community at large, except as an undefined concept without any reference to structural entities, and this time is really not very far back. It is enough to go back to 1952, when Hodgkin and Huxley published their study on the ionic basis of the action potential of the squid giant axon due to the early inward Na⁺ current, followed by the outward K⁺ current ((Hodgkin & Huxley 1952); reviewed in (Catacuzzeno & Franciolini 2022)). They carefully characterized these currents and thoroughly studied their time and voltage dependence of activation and inactivation. Yet, in the 100-plus pages they wrote to recount their study, illustrate their hypothesis, and show the modeling results of the action potential, they never mentioned the word "ion channels." When imagining how ions would cross the membrane, the undefined concept of ion channels could not be taken into account back then; carriers were more popular for the purpose.

Things slowly changed over the next couple of decades, especially in the 1960s, when several clues emerged in favor of ion channels (reviewed in (Catacuzzeno et al. 2025)). Bertil Hille and Clay Armstrong were perhaps the ones who mostly contributed to lead the scientific community to accept the idea of ion channels as we know them today. Over the full spread of the 1960s, they were able to confirm through biophysical studies previous pharmacological observations with tetraethylammonium (TEA) and tetrodotoxin (TTX) (Tasaki & Hagiwara 1957; Narahashi et al. 1964; Armstrong & Binstock 1965)) that the Na^+^ and K^+^ permeation pathways were separate entities. Hille and Armstrong went much further, suggesting that they were protein pores of different sizes and selectivity. They began calling these pores "channels" at a time when the scientific community was rather reluctant about this. They suggested that these pores for the passage of Na^+^ and K^+^ were proteic in nature and lined by oxygen dipoles that would establish hydrogen bonds with the permeating ions (Hille 1968, 1971, 1973; Bezanilla & Armstrong 1972), in a manner similar to Mullins’ cages or membrane binding sites proposed to facilitate ion dehydration and passage through the membrane (Mullins 1961).

Later that decade, (Bean et al. 1969) and (Hladky & Haydon 1970) showed that when doping artificial bilayer membranes with certain antibiotics or proteins, discrete, step-like currents of a few picoamps were observed and interpreted as due to the insertion of individual pore-like structures in the membranes. Caution was due in this case because these observations were obtained on artificial membranes. Similar unitary conductance was, however, estimated for the postulated ion channels in living cells when the maximal Na^+^ currents determined in electrophysiological experiments were divided by the number of TTX binding sites per unit area of nerve and muscle, assuming that these corresponded to the number of the postulated Na^+^ channels (due to the high selectivity of TTX for Na^+^ channels and one-to-one stoichiometry (Almers & Levinson 1975; Levinson & Meves 1975; Narahashi 2008).

Similar estimates of the conductance of individual acetylcholine (ACh)-activated receptor channels also derived from measurements of the spontaneous fluctuations (noise) of ACh-activated currents at the frog neuromuscular junction following the application of the agonist (Katz & Miledi 1972; Anderson & Stevens 1973). Using a simple physical model in which single ACh channels open and close randomly, as channels were expected to do, from these fluctuations they were able to estimate a single-channel conductance around 20 pS. These estimates were congruent with the elementary currents recorded with antibiotics and proteins inserted in artificial membranes and with the conductance estimated from macroscopic Na^**+**^ currents divided by the TTX-estimated binding sites. Confidence was thus growing that ion channels were in fact present in the membrane of living cells and that single-channel currents would be eventually recorded from them. However, as Armstrong himself later admitted, there was still no compelling evidence for the presence of ion channels in cell membranes in the early 1970s (Armstrong 2007). At the time, many believed that conclusive evidence of the presence of ion channels in the membrane would come from recording their unitary currents. This became the ultimate frontier in electrophysiology.

## First Single-channel Recording

What became immediately apparent was that in order to record the currents through individual channels, it was necessary to delimit a small area of the membrane in which there would only be one or a limited number of active channels whose individual currents could be identified. Recordings of discrete, step-like ‘single-channel currents’ of a few picoamps, supposedly passing through the postulated membrane channels, were not possible at the time in living cells because of the excessively high background noise (estimated to be at least one order of magnitude higher than the single-channel currents measured in membrane bilayers or estimated through noise analyses).

Meanwhile, experiments were conducted to record elementary currents through single channels using purified proteins in artificial lipid membranes. These experiments focused on ACh receptors and Na⁺ channels. Unfortunately, these efforts also failed because the proteins used were not fully functional. However, they provided valuable insights when studying gramicidin A. This small peptide is a perfect model for these early studies because it spontaneously forms a transmembrane channel by dimerizing in the bilayer. Experiments with gramicidin A revealed discrete, random current steps, proving that ions pass through gated pores rather than flowing continuously through the membrane matrix (Bamberg & Läuger 1973; Hladky & Haydon 1973).

This crucial scientific objective of recording single-channel currents apparently fraught with insurmountable problems found a solution when it crossed the paths of two young German scientists, Erwin Neher and Bert Sakmann. Erwin Neher had returned to Germany in 1967 after a year on a Fulbright studentship at the University of Wisconsin, where he studied spectroscopy of macromolecules, and was now looking around for some PhD project in biophysics, possibly related to nerve excitation. This search brought him to Prof Dieter Lux’s laboratory at the Max-Planck-Institut für Psychiatrie in Munich to study synaptic mechanisms in snail motor neurons. To avoid space clamp problems, Lux recommended that his young postdoc use suction pipettes to record currents. This suggestion would be recalled a few years later when trying to record elementary currents through single-channel.

While at Prof Lux’s laboratory, Neher met Bert Sakmann who had come to learn the basics of voltage clamping synaptic currents in snail neurons before moving to University College London to work in Bernard Katz’s biophysics laboratory on that topic. Sakmann was eager to understand the fundamental mechanisms of the synapse that Neher was studying because he believed they were essential to comprehending the central nervous system’s excitability. For this reason, they had many lively discussions. Following his interests, Sakmann spent three years (1971–1973) at University College in London. There, he began to think that single-channel currents could be recorded directly from cell membranes while working in Bernard Katz’s department. It was in that period that Katz, in collaboration with Ricardo Miledi, suggested that elementary current events through individual ACh receptor channels caused the noise observed in the membrane potential recordings when ACh was applied to the end-plates of frog skeletal muscle (Katz & Miledi 1972).

Neher and Sakmann met again at the Max-Planck-Institut für Biophysikalische Chemie in Göttingen in 1973. Neher had joined the Institute to gain expertise in single-channel recording in artificial lipid bilayers, and Sakmann had returned from London and was offered by Prof Otto Creutzfeldt to run his independent laboratory. Given their clear communion of interests, they soon agreed on collaborating to measure single-channel currents.

### Prologue

Neher and Sakmann knew that to record currents through individual channels, they had to electrically isolate a small area of the membrane containing a limited number of active channels. They tried to demarcate these areas by pressing fire-polished glass pipettes with an aperture size of 1–2 μm onto the membrane surface. Denervated muscle fibers were used for the experiments, which Sakmann was very familiar with from his work in Katz’s laboratory in London, particularly the enzymatic procedure for cleaning the cell surface. Despite repeated trials under different conditions, they could only achieve seal resistances of 50–100 MΩ. Although these seal resistances were modest, they were sufficient to convey a significant fraction of the current coming through the isolated patch into the recording pipette and to lower the background noise generated by ions passing through the seal resistance to an acceptable level.

**Fig. 1:**
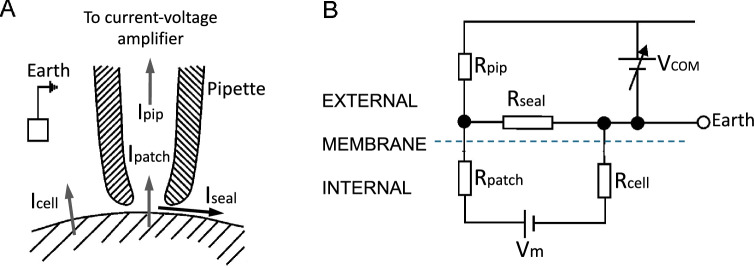
Arrangement of patch pipette and cell membrane and the equivalent electrical circuit. **A** The cartoon illustrates the relationship between the patch pipette and the cell membrane, focusing on the critical role of seal resistance in determining the fraction of current that passes through the patch pipette to the current–voltage amplifier (Ipip). This current, which represents the signal of the recording, is what remains of the current passing through the active channel(s) in the membrane patch (Ipatch), after leakage through the seal resistance (Iseal). Icell is the current through the cell membrane other than the patch. This current is generally negligible because the voltage drop commanded experimentally falls virtually entirely on the patch membrane due to its much higher resistance compared to the rest of the cell membrane. **B** Equivalent electrical circuit of the arrangement between the patch pipette and the cell membrane shown in A). Note the crucial node where the current through the patch is divided between Rpip and Rseal in reverse proportion to their sizes. In other words, the bigger the seal resistance, Rseal, compared to the pipette resistance, Rpip, the bigger the current that will flow through the patch pipette and the stronger the signal it will bring to the current–voltage amplifier. [Modified from (Ogden & Stanfield 1994)]

These aspects can be appreciated by looking at the resistances involved in the establishment of the mechano-electric seal between the membrane and the recording pipette (Fig. 1A). Of the various sources of electrical noise in the patch clamp recordings, the one resulting from the seal resistance is the most important. This noise, known as thermal noise, results from random motion of thermally excited charges through a generic resistance. It is quantified by the following general relation,

1 $$\sigma_{n} = \sqrt {4kTf/R}$$

where *σ*_*n*_, the root-mean-square deviation of the current (the background noise) through a resistor, which depends inversely on its resistance *R* (and directly on the bandwidth, *Δf*, at which the measurement is done). Here, *k* and *T* are the Boltzmann constant and the absolute temperature. This relationship has general validity and works for any type of charge moving through a resistive pathway, including the seal resistance (leakage path) between the glass pipette and the membrane.

In this regard, it is important to recall that current noise is generated by all the resistances encountered by the current from the ground to the amplifier input. These resistances include the cell membrane resistance, the patch resistance, the seal resistance, and the electrode access resistance (Rcell, Rpatch, Rseal and Rpip in Fig. 1B). However, since the seal noise appears to be, with regard to this discussion, the most important component of the overall noise of the recording system, we will briefly continue to dwell on it with a quantitative example.

To estimate the current noise that would originate from the seal resistance and determine how this would impact on single-channel current recording, it is convenient to use Eq. 1 above. Given that *4kT* equals 1.6 × 10^−20^ Joules and that typical recordings are done at a filter setting of 1 kHz, a seal resistance of 100 MΩ – which was common during the initial attempts to record single-channel currents – will generate background noise of 0.40 pA (rms). However, some additional noise arising from the seal should also be considered, due to the frequency-dependent noise introduced by the capacitance of the glass pipette walls. This combined level of noise appeared rather high to record clear current events in the order of a few picoamps, as estimated by noise analyses (Almers & Levinson 1975; Levinson & Meves 1975) or by doping artificial bilayer membranes with antibiotics or proteins (Bean et al. 1969; Hladky & Haydon 1970).

### First Single-channel Recording

**Fig. 2:**
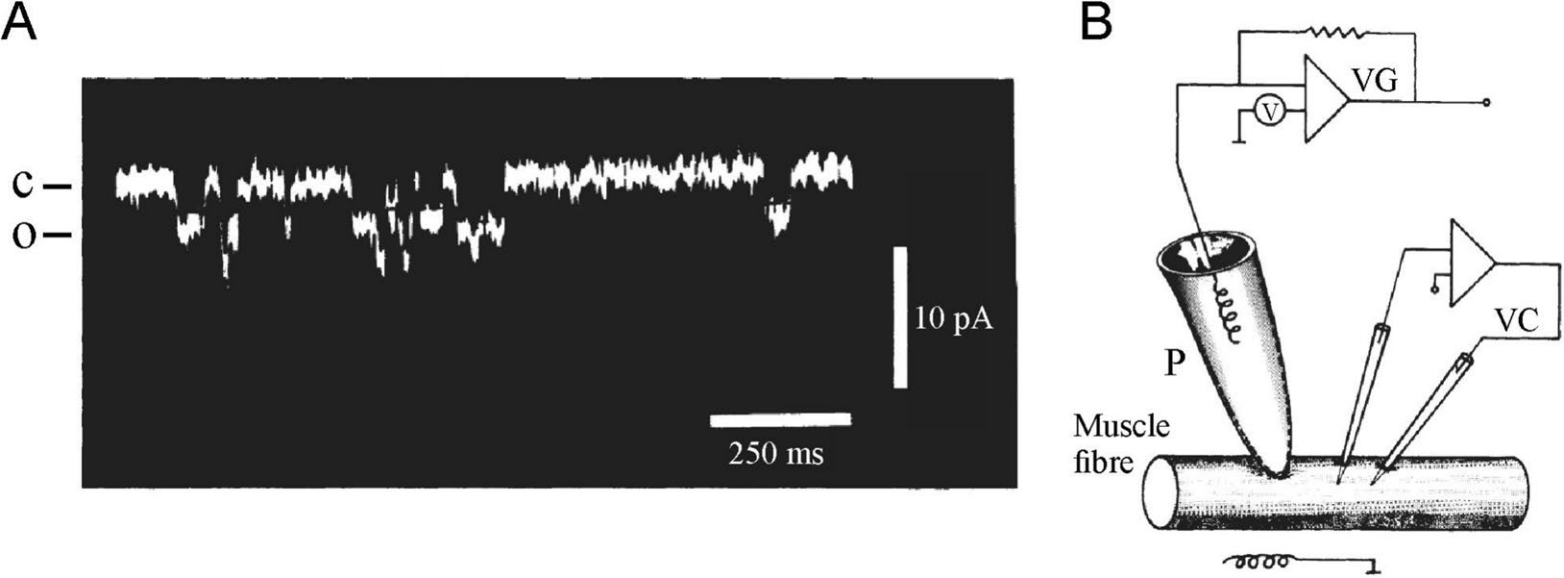
First single-channel recording from frog muscle. **A** Oscilloscope recording of single-channel current through ACh receptors present in the membrane patch from a denervated frog muscle fiber. The receptors were activated by 200 nM suberyldicholine, a more effective ACh analogue, which was placed in the patch pipette. Membrane potential was set at − 120 mV. C and O denote the closed and open levels, respectively. Note few brief double openings (not indicated). **B** Recording setup: It consisted of the denervated muscle fiber and the patch pipette (P), as well as the circuit diagrams for amplifying and recording the single-channel currents (VG) and the classic two-microelectrode voltage clamp system (VC), which controls the membrane patch potential. The patch pipette had an opening of 3–5 µm and a resistance of 2–5 MΩ. [Reproduced from (Neher & Sakmann 1976)]

In 1976 Neher and Sakmann reported the first recordings of discrete, step-like currents from a membrane patch of a denervated muscle fiber perfused with ACh (Fig. 2A) (Neher & Sakmann 1976). These were immediately interpreted as single-channel currents through the ACh receptor. Thus, they were considered conclusive evidence of the presence of ion channels in native membranes. In addition to the many lines of indirect evidence accumulated over the past decade and the recently proposed membrane model of (Singer & Nicolson 1972), which included integral proteins spanning the full membrane, these step-like currents occurring randomly with random current event lengths were perfectly congruent with the notion of ion channels that open and close in an all-or-none, stochastic manner.

These recordings, including those much better resolved, obtained with the gigaseal improvement (see below) *“conclusively proved the existence and function of ion channels”,* as stated by the Stockholm Committee when awarding the 1991 Nobel Prize in Physiology or Medicine to Neher and Sakmann for their studies. After recording single-channel currents, the general sentiment was that now channels could be seen to switch back and forth, from closed to open, instead of being assumed, as before the single-channel recordings.

However, these recordings showed substantial noise, primarily due to the poor seal between the glass pipette and the muscle membrane, which resulted in current dispersion through it. This situation, which continued as such until the end of the 1970s, did not allow for rigorous characterization of the channels’ biophysical properties. However, it immediately demonstrated the technique’s extraordinary potential in enabling real-time observation of conformational transitions in single channel in their native environments when they open or close in response to specific stimuli, such as changes in membrane potential, specific agonists, or mechanical stimuli.

It should be added that in those years that Neher and Sakmann were refining the technique to record elementary currents from muscle fibers, Neher spent good parts of 1975 and 1976 working as a postdoc in the laboratory of Charles (Chuck) Stevens at Yale. There, under his watch, Neher conducted experiments similar to those done in Göttingen, obtaining similar results. In fact, the first single-channel recording may even have been made at Yale. Stevens was certainly closely involved with this new technique if he was asked to coauthor the 1976 Nature paper (Zador et al. 2023). In any case, Neher did acknowledge the work done at Yale, particularly the single-channel recordings conducted there, which he regarded as a crucial reassuring proof, having been made in a different laboratory with a completely different set-up.

### Gigaseal Formation: A Breakthrough in Patch Clamping

The excessive noise of the first single-channel recordings had to be reduced drastically if meaningful studies on ion channels were to be carried out. Neher and Sakmann knowingly focused on the seal resistance. They tried to improve cell surface cleaning to achieve a better seal and to coat the pipette tip to reduce capacitance, which is another source of noise. They also tried different types of pipette glass with different charges, but none of these changes resulted in major improvement. In the meantime, they had assembled an excellent team of scientists in Göttingen, including Owen Hamill, Alan Marty, and Fred Sigworth. This team turned out to be extremely creative, providing innovative ideas for technical improvements to the method, offering theoretical insights into how channels work, and developing sophisticated analyses of single-channel recordings.

Disappointed by continued failures, Neher, Sakmann, and the others were on the verge of abandoning any further attempts to reduce seal resistance when they noticed that high seal resistances in the range of 10 to 100 gigaohms (GΩ) would easily form by applying mild suction with a pipette (Baker 1981). These seals, in the gigaohm range, later called gigaseals to distinguish them from the earlier megaohm seals, had the crucial advantage of reducing the background noise of the recording by more than an order of magnitude, enabling single-channel currents in the picoamp range to be resolved and the membrane patch to be voltage clamped without the use of additional microelectrodes (Sigworth & Neher 1980).

**Fig. 3:**
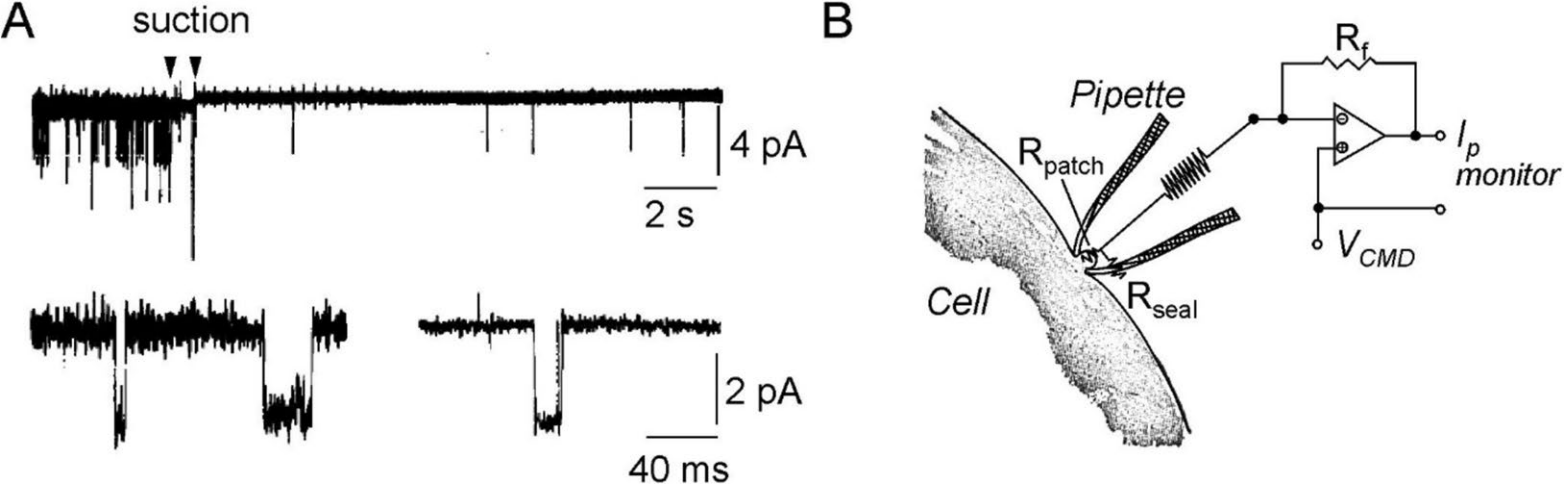
Gigaseal formation between the pipette tip and the cell membrane. **A** *Upper trace*: Continuous single-channel recording before and after application of mild suction through the patch pipette. The elementary current events are arguably from ACh receptors’ activation (note double openings at the beginning of the recording) since the patch pipette contained suberyldicholine (100 nM). At the beginning (before the first arrow), the seal resistance between the patch pipette and the membrane, attained by only pressing the pipette against the membrane, was about 150 MΩ. Following slight suction through the patch pipette (applied between the two arrows), a gigaseal of about 60 MΩ resistance formed, and background noise decreased markedly in parallel. The clear decrease in channel activity following the gigaseal formation was ascribed to transient depletion of the agonist at the pipette tip as a result of suction. *Lower traces*: Single-channel currents at higher magnification before (*left*) and after (*right*) the formation of the gigaseal. All recordings were carried out at the resting potential of the cell. Filter setting was 1 kHz for the upper trace and 3 kHz for the lower traces. [From (Hamill et al. 1981)]. **B**) Schematic electrical circuit of the patch clamp and the headstage current/voltage amplifier showing the major resistances involved. Since the pipette current, which originates from the patched membrane and consists of what remains after leakage through the seal resistance, passes through the feedback resistance of the inverting amplifier, Rf, using large values of Rf minimizes the thermal current noise. This is important for resolving minute currents during single-channel recordings. [Modified from (Sigworth & Neher 1980)]

The marked decrease in background noise made the single-channel current recordings much better resolved, as shown in Fig. 3A from the comparison before and after the formation of the gigaseal. The upper trace shows the time course of the development of a gigaseal in an enzymatically treated frog muscle fiber, that brings the seal resistance of about 150 MΩ when the tip is only pressed on the muscle surface to about 60 GΩ attained upon applying the negative pressure (between arrows). The reduction of background noise can be clearly seen by comparing the baseline thickness (when no channel is active). The lower recordings show single-channel currents on an expanded timescale and at higher resolution before (*left*) and after (*right*) formation of the gigaseal, as well as the marked reduction of background noise.

A clear benefit of high seal resistance is that more of the membrane current flows into the pipette instead of passing through the glass/membrane seal. As a result, the signal-to-noise ratio increases (cf Fig. 3B). However, a more important aspect of high seal resistance is the parallel reduction of the background noise originating from the thermal motion of ions through the seal resistance (cf. Equation 1). For example, with gigaseals of 10 GΩ, which are routinely attained today, we can estimate a background noise of *σ*_*n*_ = 0.04 pA (rms) from Eq. 1. Compared to the initial experiments, when the seal resistance achieved was only about 100 MΩ and *σ*_*n*_ = 0.4 pA (rms; still estimated from Eq. 1), there is a tenfold reduction of background noise.

#### The Physico-chemical Basis of Gigaseal Formation

The physical and chemical aspects of how gigaseals form remain unclear, despite the extensive research conducted in this regard. However, these studies have identified important factors and conditions that favor successful gigaseal formation. Van der Waals forces are thought to be the main forces involved in sealing the interior patch pipette and the membrane, consistent with their chemically unspecific nature and the observation that gigaseals form with different cell types as well as with pure lipid membranes (Parsegian 2006). The formation of the gigaseal has been suggested to result from van der Waals forces squeezing out the interposed water layer between the membrane and pipette glass. This occurs when the seals are in the hundreds of megaohm range and the membrane and glass are 20–50 Å apart (Nir & Bentz 1978; Parsegian et al. 1979). When the seal resistance approaches values in the order of 10 MΩ, the estimated distance between the patch membrane and the pipette glass is only a few angstroms. Evidence supporting the close contact between the membrane and the glass without interposed water includes the observation that small molecules cannot cross the gigaseal. For example, ACh fails to activate ACh receptors in the patch when placed in the bathing solution of a gigasealed patch membrane.

### The Classic Configurations of the Patch Clamp and Their Use

The formation of the gigaseal not only lowers greatly the noise on the recordings, it also renders the pipette/patch interaction mechanically very strong and amenable to various sorts of manipulations that allow one to attain different patch configurations, each very versatile and useful for addressing specific scientific questions.

#### The ‘Cell-attached’ Configuration

**Fig. 4:**
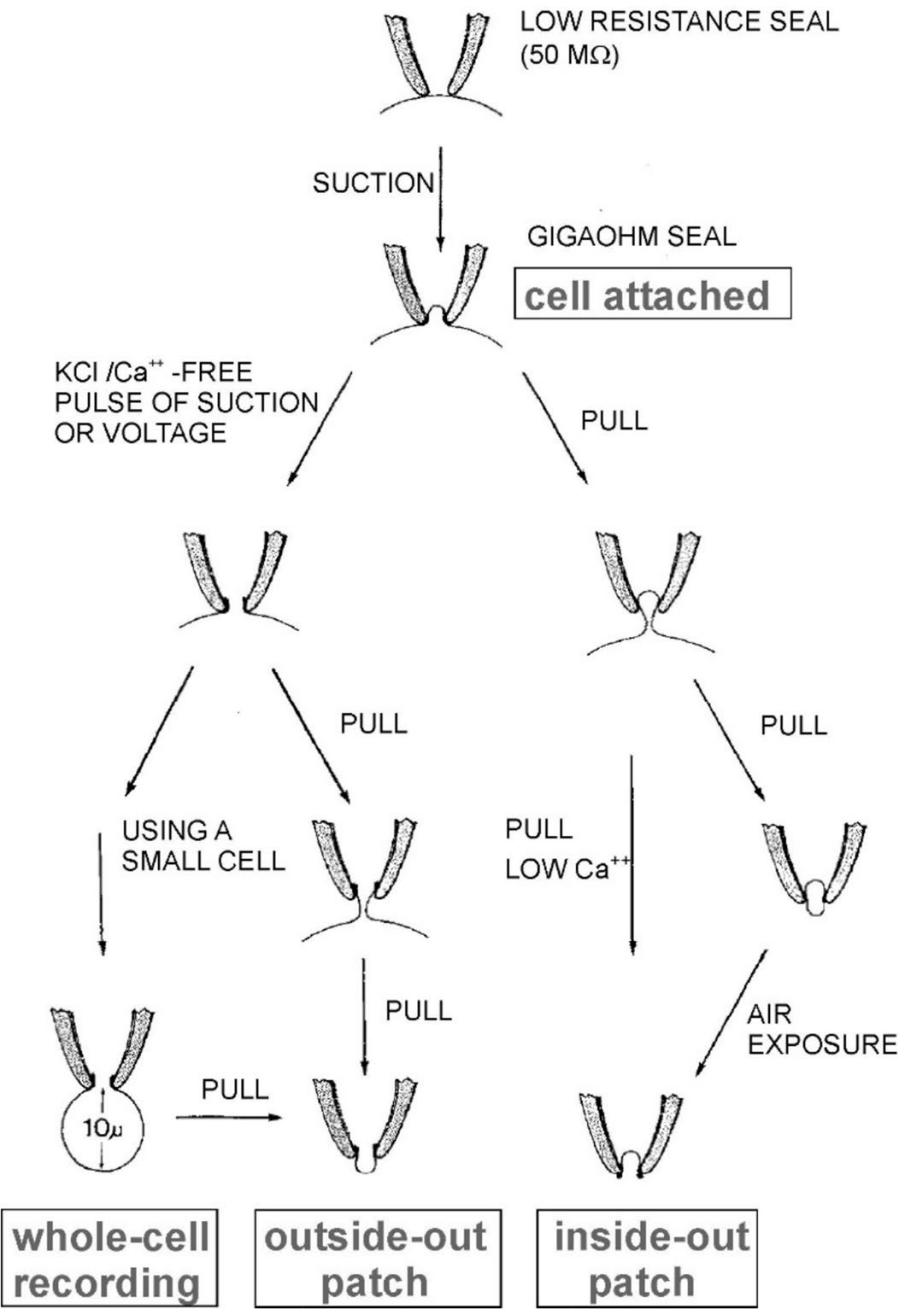
Schematic illustration of the four different configurations of patch clamp (indicated) and how they are obtained. See text for description. [From (Hamill et al. 1981)]

The formation of the gigaseal obtained by applying a moderate negative pressure inside the patch pipette once it has been lowered to touch the cell membrane establishes the initial configuration referred to as ‘cell-attached’ for obvious reasons (Fig. 4, indicated). It is also the precursor configuration to all other variants of the patch clamp technique. This configuration is very stable and allows measurement of the unitary currents through the channel(s) located on the dome of the membrane patch sucked inside the pipette. It is possible to record only the unitary current through the channel(s) in the patch when there is a second membrane, the remaining cell membrane, between the two electrodes because this second membrane’s resistance is extremely low compared to the patch resistance and can be safely ignored. However, this configuration is greatly limited because it is not possible to change the solutions (i.e., the type as well as the concentrations of ions or other compounds) on either side of the patch membrane. Moreover, given the much higher resistance of the patch membrane compared to the rest of the cell membrane, with this configuration it is not possible to control the membrane potential of the cell (but only that of the membrane patch).

#### The ‘Inside-out’ Configuration

Both these limitations are bypassed by quickly withdrawing the patch pipette from the cell after obtaining a cell-attached configuration. This maneuvre causes the membrane patch inside the tip of the patch pipette to be torn from the cell while maintaining a gigaohm seal with the pipette and mechanical stability. This configuration is referred to as ‘inside-out’ because the inside of the patch is now exposed to the bath solution. Noteworthy, this configuration allows the solution perfusing the cytoplasmic (inside) face of the patch membrane to be changed according to experimentation needs (Fig. 4, indicated). The inside-out configuration is appropriate for studying modulation of ion channels by agents or compounds that interact with the cytoplasmic side of the channel, such as modulation of Ca^2+^-activated K^+^ channels by Ca^2+^ ions. Furthermore, this configuration, in which there is only the patch membrane between the two electrodes, allows the control of the membrane potential to be accurate. Note that this configuration, on the other hand, is of no help in studying channel activation by neurotransmitters or, in general, any kind of modulation that occurs through binding sites located on the outer face of the membrane, for the reason that it is not possible to change the solution inside the patch pipette. Note that a vesicle sometimes forms at the end of the tip to attain this configuration. This vesicle can be broken by briefly exposing it to air. Using low Ca^2^⁺ solutions can be an alternative, as they have been suggested to reduce vesicle formation.

#### The ‘Whole-cell’ Configuration

If, instead of withdrawing the patch pipette from the cell after obtaining the cell attached configuration, a stronger negative pressure is applied to the patch pipette, the patch inside is ruptured thereby bringing the intracellular milieu in contact with the solution inside the pipette. This configuration, called ‘whole cell’ configuration, unlike all the others presented above, allows one to study macroscopic currents originating from all the channels present on the entire membrane of the cell (Fig. 4, indicated). An additional feature of whole cell configuration, which can be regarded either as an advantage or disadvantage, depending on the aim of the experiment, is that compounds easily diffuse from the patch pipette into the cell and their effects on ion channel function can be studied. However, at the same time, the pipette solution eventually replaces the cytoplasm of the cell and all its important factors, which are washed out through the patch pipette.

#### The ‘Outside-out’ Configuration

The whole cell configuration is not only an interesting way to study macroscopic currents with low-resistance electrodes. It also represents the intermediate step to the last classical patch clamp configuration, the ‘outside-out’ configuration. By slowly withdrawing the patch pipette from the cell, in the whole cell configuration, an elongated neck is initially formed, which rapidly collapses and then separates from the cell (Fig. 4, indicated). This configuration, like the inside-out configuration, has the patch exposed to the bath solution, which can now be changed as needed. Yet, it differs from it because the side of the patch now exposed to the bath solution is the extracellular portion of the membrane. This has interesting experimental properties because it allows one to study the effects of extracellular modulators of ion channels. The mind immediately goes to the myriad of neurotransmitters and associated ionotropic receptors that this configuration allows one to study. The drawback of this configuration is the much more laborious process to obtain it and its moderate stability.

#### Other Configurations of the Patch Clamp

Over the years, other patch clamp configurations have been developed, including the “perforated patch”, “loose patch” and “giant patch”. However, since they are all meant to record macroscopic currents, we will not describe them further. Readers interested in learning more can find descriptions of these configurations in the following papers (Pusch & Neher 1988; Rae et al. 1991; Roberts & Almers 1992; Stühmer 1992; Collins et al. 1992).

## Analysis of Single-channel Recordings

Single-channel recording provides crucial information about ion channels and their behavior. This section briefly illustrates what can be measured from the experimental recordings of single-channel currents and what information can be drawn. There are two immediate measurables in them: the amplitude of the current and the time spent in the open and closed states.

### Constructing Amplitude Histograms

Measuring the amplitude of the elementary current through single channel can be of interest as a criterion for characterizing or identifying channels. Moreover, when current (conductance) measurements are made with different ion solutions, different membrane potentials, information on the mechanism of ion permeation and selectivity is obtained.

The most direct way to measure the current amplitude of an open channel is to make a point-amplitude histogram by plotting all the digitized current values of the single-channel recording. In the presence of only one active channel, this will produce a peak at the closed level and a peak at the open level, unless the channel has subconductance states (in which case there will be as many peaks for the open channel as there are conductance states). Another piece of information that can be derived from the point-amplitude histograms is that the area under each peak is proportional to the time spent in the corresponding state. This means that the open probability of the channel under the given experimental conditions — that is, the fraction of time the channel is in the open state — can be calculated immediately.

### Kinetic Analysis of Single-channel Recordings

More informative is analyzing the single-channel recordings to extract information on the channel’s kinetics, i.e., the underlying changes in channel conformation and gating. The recorded single-channel currents are ideally square pulses of random durations that reflect the stochastic and virtually instantaneous switching of the channel between open and closed states. Under steady-state conditions (i.e., constant voltage or agonist concentration for voltage- or ligand-gated channels, respectively), the channel switches between several possible states at a constant rate.

**Fig. 5:**
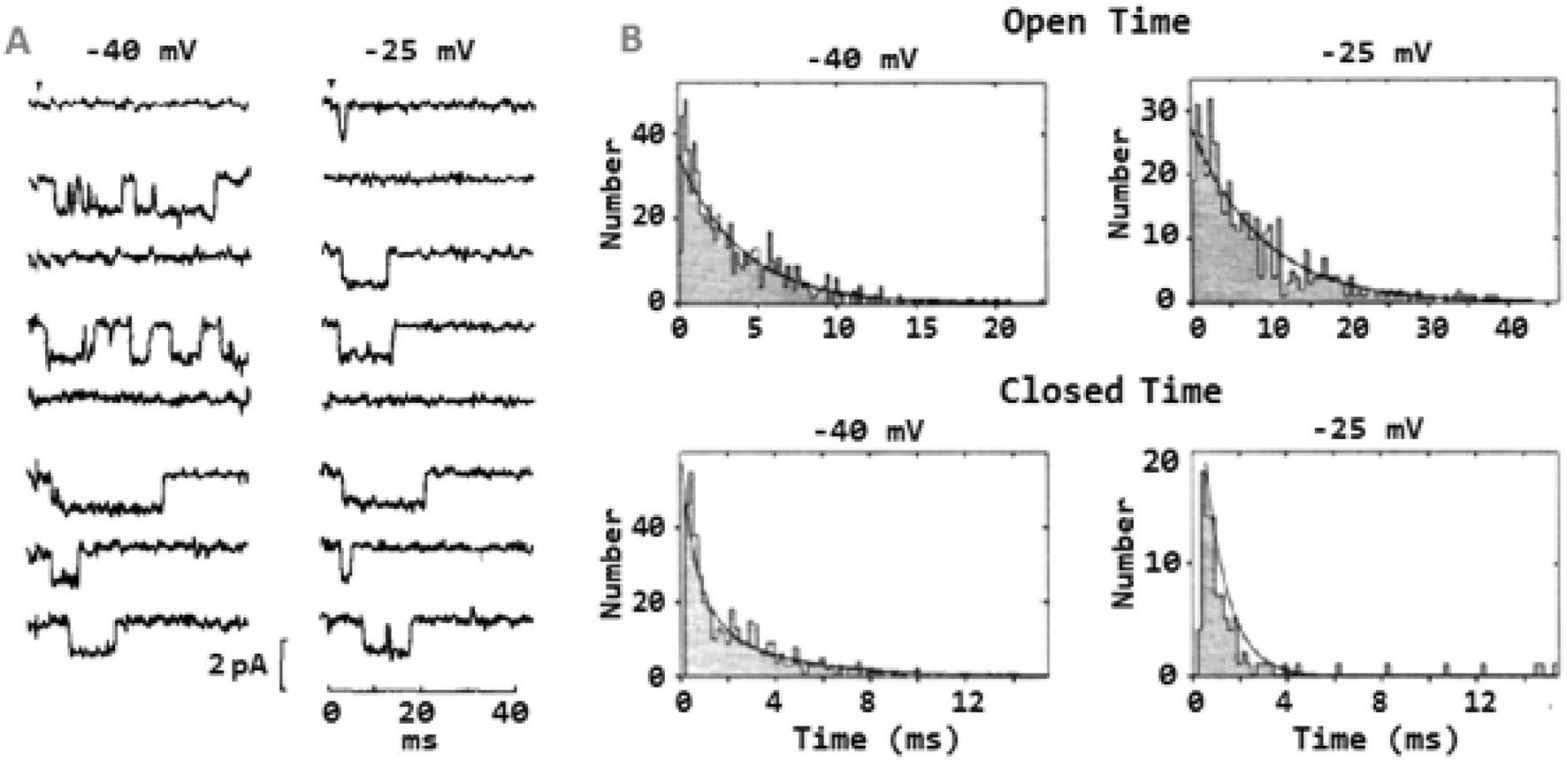
Single-channel recordings and dwell-time distributions of the open and closed times from voltage-gated Na^+^ channels. **A** Single-channel recordings in outside-out configuration from a patch of cultured GH_3_ cells containing only one active voltage-gated Na^+^ channel. Patches were subjected to voltage steps (starting at the arrow) to -40 and -25 mV from a holding potential of -120 mV. **B** Distribution on linear axes of open and closed times at -40 and -25 mV. The solid lines are the best fit of dwell-time distributions. Both dwell-time distributions of the open state were well fitted with a single exponential, whereas three exponentials were needed to satisfactorily fit the dwell-time distributions of the closed state, suggesting that the channel can enter three closed states. [From (Horn & Vandenberg 1984)]. Note that the dwell-time histograms shown here are plotted in the linear form, which results from splitting the time axis into bins of equal size. However, Blatz and Magleby have demonstrated the double utility of plotting histograms on a logarithmic time axis (Sigworth & Sine 1987; Blatz & Magleby 1989). First, dwell times falling over several orders of magnitude can be compactly represented in a single plot, instead of several, as shown in Fig. 6. Second, the time constant of each exponential can be identified more easily, since it is represented by the maximum height of the fitted function

These state transitions form the basis of classic kinetic analysis methods, which involve measuring the duration of each open and closed event in a current recording. Notably, this type of analysis, for which the basic techniques had already been established by earlier studies on gramicidin A (Bamberg & Läuger 1973; Hladky & Haydon 1973; Zingsheim & Neher 1974), soon revealed an essential property of ion channels: the distribution of dwell times in the closed and open states was typically well described by exponential functions. Figure 5 illustrates this for an early set of single-channel recordings of the voltage-gated Na^+^ channel from cultured GH3 cells, showing that the distributions of open events at varying potentials are well described by single exponentials (Fig. 5B, upper plots). By contrast, a three-exponential function is needed to fit the distributions of the closed events (Fig. 5B, lower plots) (Horn & Vandenberg 1984).

The multiplicity of exponentials is a common occurrence, especially in dwell-time distributions of channel closures. They supposedly originate from several transitions occurring over a sequence of closed conformations (states) before the channel opens. This occurrence poses a significant challenge to the kinetic analysis of single-channel recordings when attempting to determine the number of states because transitions between closed (or open) states cannot be detected by changes in current level. In these cases, the minimum number of states that the channel passes through before opening corresponds to the number of exponential functions required to fit the closed dwell-time distributions, as the theory of channel kinetics shows. To better understand this, we first need to cover some preliminary aspects, such as the procedures for idealizing recordings, the problem of missed events, and plotting dwell-time histograms.

#### Idealization of Single-channel Records and the Problem of Missed Events

One serious problem that can bias this type of analysis, i.e., determining the precise channel dwell times, is the combination of generally small recorded currents and substantial background noise. To address this problem, idealization algorithms have been developed that transform noisy, single-channel recordings into a sequence of clean square steps of defined amplitudes and durations. From this series, one can obtain a list of dwell times for all closed and open events. These dwell times are then used to construct dwell-time histograms. This series of square steps is intended to be the best approximation of true channel activity.

Early studies typically used the 50% amplitude threshold method. This method involves placing an amplitude detector at 50% of the unitary current to detect transitions between the closed and open states (Colquhoun & Sigworth 1983; Sachs 1983). Although intuitively sound and conceptually simple, this method introduces distortions due to the heavy filtering that is usually necessary to distinguish closed and open events unambiguously. Heavy filtering poses the challenge of missing short events because they do not have enough time to reach the 50% threshold, due to the finite time response of the low-pass filter. This problem is compounded by the poor frequency response of the patch clamp system and the noise level of the recordings. The consequences are often substantial. Missing a short closure not only increases the mean value of the distribution of closed dwell times, but also increases the distribution of the open dwell times. This occurs because it lengthens the opening times of the events during which the missed closures occurred. Addressing this problem was generally not easy. Magleby and his coworkers were among the first to develop methods dedicated to correcting for missed events in models with multiple open and closed states connected in various ways (Blatz & Magleby 1986; Magleby & Weiss 1990). However, it was only after a proper solution to the problem was found that a comprehensive approach to dealing with missed events became possible (Hawkes et al. 1990, 1992; Colquhoun & Hawkes 1996).

Current fluctuations (noise) in single-channel recordings can be the source of a similar problem in that they introduce artifactual events (events that do not exist) into the idealized recordings due to excessive fluctuations (noise) in the current. In contrast to missing short openings, which increase the mean dwell times of both closed and open events, the artifactual introduction of short (so they are) openings reduces the mean dwell times of both openings and closures. This is because it introduces very short open events and cuts a longer closure into two shorter ones, during which the artifactual opening occurs. The same reasoning applies to the artifactual introduction of closed events.

Because it is often difficult to achieve a satisfactory idealization of single-channel recordings, this problem is bypassed altogether when addressing single-channel kinetics by using hidden Markov modeling (HMM). This approach analyzes noisy data directly, eliminating the need for idealization. Compared to threshold crossing, this method considerably improves detection efficiency, especially with poor signal-to-noise ratios. We will revisit hidden Markov modeling shortly, once we have introduced the basics of the Markov model and its properties.

### The Kinetic Behavior of Channels is Described by Discrete-State Markov Models

Early analysis of single-channel recordings soon showed that the kinetic behavior of ion channels can be well described by discrete-state Markov (DSM) models (Colquhoun & Hawkes 1981). These models are represented by kinetic schemes that typically include multiple closed and open states connected by rate constants and display the following basic properties: *i)* conformation changes (transitions) between any two states are virtually instantaneous, i.e., the time required is negligible compared to the time spent in either state; *ii)* transition rate constants between stable states do not vary under constant experimental conditions; *iii)* the dwell times of each state are distributed exponentially, and the number of exponentials required to fit the distributions of closed and open dwell times represents the minimum number of closed and open states of the channel; *iv)* at any given moment, the channel has no memory of which conformation it came from or how long it has been in its current state.

For DSM models, as well as the ion channels they are thought to describe, the dwell times in any given state are random (stochastic) variables whose distribution can be described by the following exponential function, *f(t)*, such that

2 $$f\left( t \right) = \left( {{1}/\tau } \right){\text{exp}}^{( - t/\tau )}$$

where *t* is time, *τ* is the time constant of the exponential distribution, and 1/*τ* is the value of the distribution at time 0. (Note that the product of *τ* by the value of the exponential at time 0 is the area under the exponential curve. This is an important parameter for comparing the relevance of each exponential, i.e., each state.) This analysis demonstrates that the exponential distribution of dwell times in each state is mathematically expected from Markov properties. It also demonstrates that Markov models can be applied to ion channels.

A more intuitive way to illustrate why the dwell times of ion channels (and DSM models) are exponentially distributed is to consider the simple case of a channel with two states, open and closed. According to the kinetic rate theory, on which DSM models are based, once the channel enters one of the two states (e.g., the closed state), it must overcome the energy barrier separating the two lower-energy states in order to return to the open state. In microscopic thermodynamics, these jumping efforts are viewed as the channel’s repeated attempts to overcome the barrier using the (varying) thermal energy available at each attempt. Assigning a specific probability to each attempt by the channel to overcome the barrier makes it easy to see that the probability is highest on the first attempt and decreases exponentially with each successive attempt. This is because a channel that crosses the barrier on the second or third attempt requires that the channel has failed to cross the barrier on the first attempt, or on the first and second attempts combined. Thus, there are exponential distributions of channels’ dwell times.

### Analyzing Single-channel Data

From the previous discussion, it can be concluded that single-channel recordings can be analyzed using Markov modeling. In these models, each state indicates a distinct conformation of the channel. The problem arises when one must decide how many states to include in the model, i.e., how many significant conformations the channel has and how they are connected. This point is not trivial because, as mentioned, ion channels often exhibit several conformations having the same conductance. These conformations are often difficult to identify because their number can only be derived from the number of exponentials needed to fit the dwell-time distributions constructed from single-channel recordings. As we have seen, these distributions are subject to biases introduced by missed and artifactual events during the idealization procedure.

Because of the poor discrimination of the number of states and especially their connectivity that can be recovered from one-dimensional histograms, this type of analysis was improved a few years later with the introduction of two-dimensional histograms containing correlation information between adjacent events, which was not included in previous analyses (Fredkin et al. 1985; Magleby & Weiss 1990). Although increasing the dimensions of the histogram increased the amount of information that can be extracted from single-channel data, the histogram fitting approach was found to be unsuitable for obtaining the desired information. This is because it continued to suffer from the initial limitation of missed or artifactual events, especially when dealing with low signal-to-noise conditions.

To overcome some of these limitations, (Magleby & Weiss 1990) took a different approach and used a simulation method. This method involves iterative simulations of single-channel currents from an initial putative kinetic model and then compares the dwell-time histograms generated with those obtained experimentally. The routine also includes the continuous adjustment of the model parameters to optimize the match between the two sets of dwell-time histograms using maximum-likelihood criterion, developed by (Horn & Lange 1983), to resolve the limitations of histogram fitting.

Another advantage of this method was that the simulated and experimental currents were analyzed in exactly the same way. This meant that the dwell-time histograms derived from the simulated and experimental current recordings would suffer the same distortions due to filtering and missed events, and, in a sense, these distortions were implicitly taken care of. The simulation method had one major drawback: its huge computational load because the data had to be repeatedly simulated and idealized. Some of these problems were removed by developing the Hidden Markov Model (HMM). This approach eliminated the idealization procedure that caused various types of data distortion and poor parameter estimates, particularly under low signal-to-noise ratios, by simultaneously modeling the current signal and noise of the raw data (Chung et al. 1990).

The conceptual approach of the hidden Markov model involves calculating the probability of reproducing single-channel currents recorded experimentally, starting from a kinetic model and a set of parameters (e.g., rate constants, channel conductance, and noise level). The strategy of this method consists of recursively changing the model parameters in order to maximize the probability of reproducing the experimental data, which is calculated using the maximum-likelihood method. One requirement to apply this method is to have a plausible kinetic model to start with (Chung et al. 1990; Becker et al. 1994; Venkataramanan & Sigworth 2002).

#### More Recent Advances and Applications of Single-channel Recording

The last 30 years have seen a significant evolution in several aspects of single-channel recording and analysis. Recordings have moved from a highly specialized, labor-intensive, manual process to automated patch clamp (APC) systems, which are essential for drug discovery and large-scale screening (Dunlop et al. 2008; Obergrussberger et al. 2021). APC systems have accelerated the identification of candidate drugs for various diseases, including cardiac arrhythmias, pain, and neurological disorders (see references in (Obergrussberger et al. 2021)). On another front, single-channel electrophysiology has been combined with optical techniques, such as (single-molecule) fluorescence resonance energy transfer (FRET), to directly correlate the channel’s electrical activity with its conformational changes (reviewed by (Weatherill & Wallace 2015)). This method involves attaching fluorescent probes to different parts of the channel. When the channel opens or closes, the distance between the probes changes, altering the FRET signal. By simultaneously recording the FRET signal and the single-channel current, researchers can directly observe how the protein’s conformation changes as it gates. This provides a level of mechanistic detail that was previously impossible.

Finally, we mention the ϕ-value analysis, which is used to establish the state of a protein’s residue when the gating reaction has reached the transition state, and to dissect fast conformational transitions of channels that are represented by single reaction rates in kinetic schemes. This is often done by systematically mutating residues along the ion channel protein and calculating the ϕ value as the fractional change in activation free energy for a gating transition relative to the equilibrium free energy (Fersht 1995). For the ACh receptor, for example, where the ϕ analysis has been largely applied, the closed → open transition has been shown to be made by a concerted series of three conformational changes that starts at the agonist binding site and propagates through the protein to finally open the channel (Purohit et al. 2007).

## Single-channel Recording to Study Channel Gating and Permeability

We report here two examples of how single-channel recording has been usefully employed to understand aspects of ion channel gating and permeability. We begin with a kinetic study of the nicotinic ACh receptor of skeletal muscle, paying homage to the channel and cell model from which the first single-channel currents were recorded. This is followed by a description of the peculiar mechanism of permeation of both anions and cations through the background Cl^–^ channel of mammalian neurons, which the single-channel recording analysis unveiled.

### Testing the Gating of the ACh Receptor Through Single-channel Recordings

**Scheme 1:**
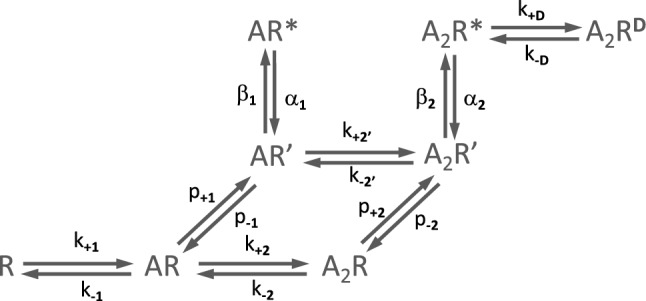
This scheme illustrates the successive binding of two ACh molecules (A) to a closed receptor (R), generating primed complexes (AR’ and A_2_R’). These then isomerise into active complexes (AR* and A_2_R*). The desensitization process is also illustrated

The first model of ACh receptor activation, proposed by (Del Castillo & Katz 1957), entailed the binding of the agonist ACh to the receptor (R). This inactive (closed) complex (AR) then isomerized into an active (open) complex (AR*) (Del Castillo & Katz 1957). Subsequent findings revealed a more complex scheme. First, the positive cooperativity of the dose–response curve implied that the ACh receptor has at least two binding sites that must both be occupied for the channel to open. A second inactive state with two bound ACh molecules (A_2_R) was then introduced before the open state (A_2_R*) (Adams 1981; Lingle et al. 1992). Second, the channel was later shown not to isomerize directly from the inactive bound states, AR or A_2_R, to the respective open states, AR* and A_2_R*. Instead, both AR and A_2_R must pass through the transient ‘primed’ intermediate states, AR’ and A_2_R’, for opening to occur (Mukhtasimova et al. 2009). Finally, it had also been shown that the channel can open with only one agonist molecule bound, though this occurs rarely and briefly (Colquhoun & Sakmann 1981; Jackson 1988). To account for these observations, the following Scheme 1 was proposed.

The Scheme also accounts for the desensitization process undergone by the ACh receptor following prolonged exposure to agonist (Cachelin & Colquhoun 1989; Dilger & Liu 1992; Auerbach & Akk 1998). This is done by adding a desensitized state, A_2_R^D^, which originates from the open state A_2_R*.

**Fig. 6:**
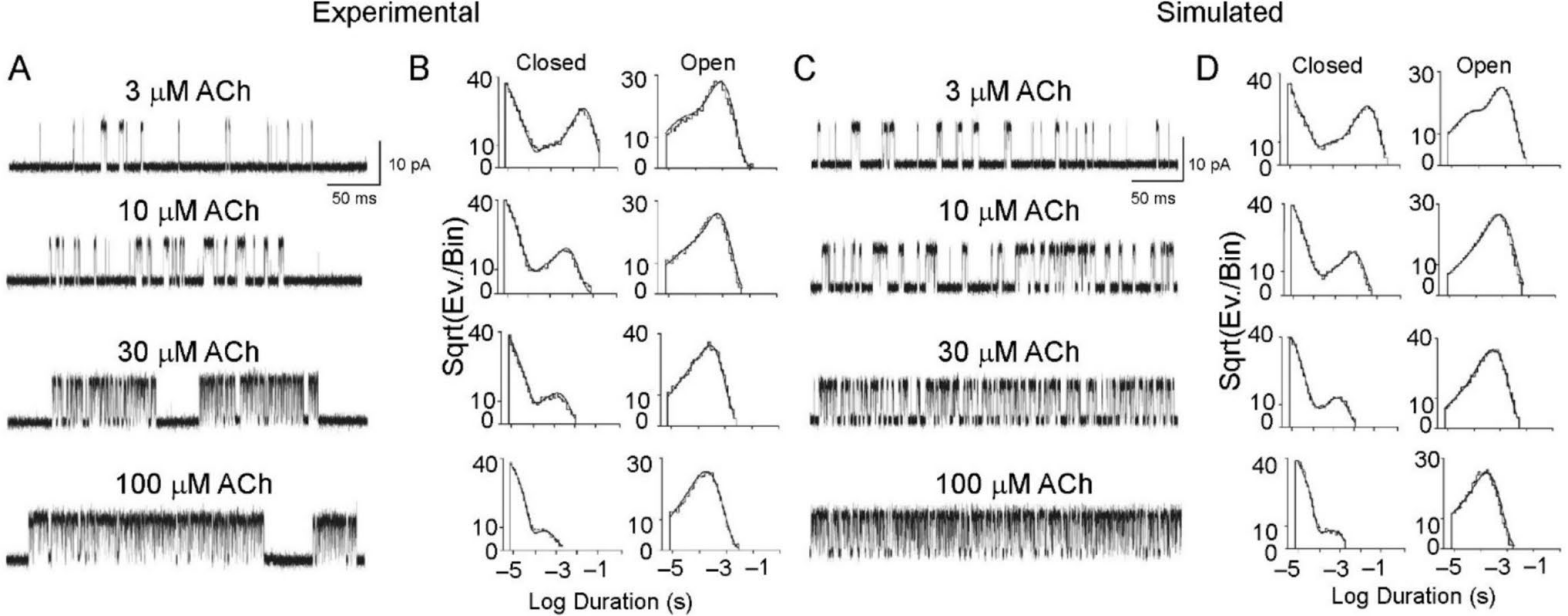
A comparison of simulated and experimental single-channel recordings from ACh receptors at increasing ACh concentrations, and corresponding closed and open time histograms. The simulated recordings were generated from the best fit of Scheme 1. The experimental recordings were obtained in cell-attached configuration at a bandwidth of 25 kHz from BOSC 23 cells transfected with cDNAs encoding for human skeletal muscle ACh receptor subunits. [From (Mukhtasimova et al. 2016)]

The scheme was complemented by rate constant estimates for each agonist binding and unbinding event, as well as each conformational transition. These estimates resulted from years of electrophysiological investigations (Sine & Steinbach 1986; Jackson 1988; Zhang et al. 1995; Auerbach et al. 1996; Wang et al. 1997) and were improved more recently due to the increased temporal resolution of single-channel recordings and analysis algorithms developed by the Sine laboratory (Mukhtasimova et al. 2016). The same laboratory then tested the congruence of the kinetic scheme with the experimental data of ACh receptors using a different strategy. This involved comparing data generated by the kinetic scheme (e.g., single-channel recordings, derived dwell-time parameters, probability density functions) with data obtained experimentally on the native ACh receptor (Mukhtasimova et al. 2016). Figure 6 shows the results of this comparison.

Both simulated and experimental single-channel recordings at increasing concentrations of ACh show very similar trends. First, there is a decrease in closed time durations and a parallel increase in channel open probability (Fig. 6A, C). These opposite trends of closed time duration and channel open probability as ACh concentration increases are reflected in the shape of the closed and open time histograms (Fig. 6B,D). In particular, the longest exponential components of the closed state gradually decrease (shifting toward the left) in both the simulated and experimental histograms. Second, the shortest open state component, present at the lowest agonist concentration (3 µM ACh), vanishes at higher concentrations, beginning at 10 µM ACh (Fig. 6B,D). Third, at ACh concentrations of 30 μM or higher, the mean open dwell time decreases compared to lower concentrations. This is likely due to the significant presence of channel-blocking events caused by the agonist at these ACh concentrations (Sine & Steinbach 1984).

The strong match between the simulated and experimental single-channel recordings and dwell-time histograms of the ACh receptor indicates that the proposed kinetic scheme — which includes agonist binding, priming of the receptor in a pre-open (closed) state, the gating step toward the open state, and the associated rate constants — well represents the kinetic behavior of the native receptor channel.

### A Single-channel Study to Unveil the Permeation Mechanism of Cl^–^ Channels

For decades, beginning with the pioneering work of Hodgkin and Huxley in the early 1950s, who accurately studied the Na⁺ and K⁺ currents responsible for cell excitation, the Cl⁻ currents have been largely overlooked (Hodgkin & Huxley 1952; Hagiwara & Takahashi 1967; Llinás & Sugimori 1980). In fact, it has long been believed that these Cl⁻ currents were unnecessary for proper cell functioning. Furthermore, by generating what were called leakage currents, which seemed to have no defined function and, in addition, presented a number of technical complexities, they were considered more of an experimental inconvenience than anything of interest.

It was only with the advent of single-channel recording that it became possible to question whether this rooted view on these currents was indeed sustainable. In the mid-1980s, when one of us arrived at Wolfgang Nonner’s laboratory at the University of Miami, he had just begun laying the groundwork for the long-forgotten Cl⁻ currents. He had started by studying their permeation and selectivity properties, using as a cell model the mouse hippocampal neuron, where these currents are present in abundance.

#### General Properties and Anion Selectivity of the Background Cl^–^ Channel

**Fig. 7:**
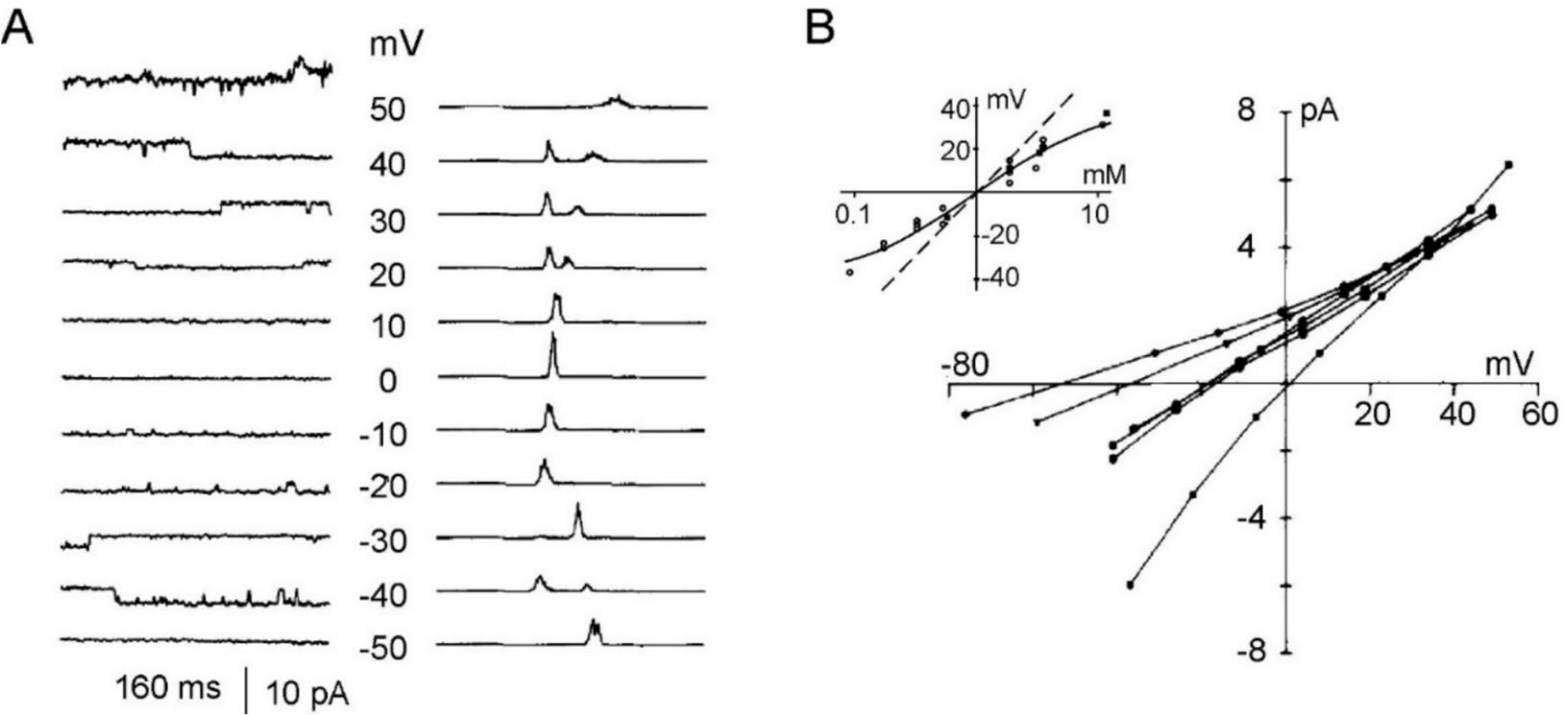
Measurement of unitary Cl^–^ currents from mouse hippocampal neurons and current–voltage relationship under varying experimental conditions. **A** *Left:* Current traces obtained from inside-out patches in symmetrical 300 mM NaCl saline, at the indicated voltages. *Right:* Amplitude histograms from the traces on the left. The abscissa of the histograms covers 10 pA. Zero current (channel closed) peaks have not been lined up. The ordinate plots the number of samples per bin. **B** Unitary currents at different potentials from an inside-out patch probed with varying NaCl concentrations (from 75 to 1000 mM) against a 1000 mM NaCl concentration in the patch pipette. *Inset*: Plot showing the relationship between reversal potentials and NaCl activity ratios across the patch. The open circles are data from individual patches while the filled squares are averages obtained for frequently used activity ratios. The straight dashed line shows the Nernst potentials for Cl^–^ ions, and the dashed curve shows the GHK voltage equation for a cation/anion permeability ratio of 0.2. [From (Franciolini & Nonner 1987)]

Background Cl^–^ channels, as they came to be known, had a single-channel conductance of about 30 pS in symmetrical 150 Cl^–^, displayed no obvious rectification and showed mild dependence on voltage (Fig. 7A,B). They were found to be poorly selective toward anions, which suggested that they had quite a wide pore. The permeability sequence, measured by reversal potentials, was Br^–^ > I^–^ > Cl^–^ > F^–^. According to Eisenman theory, this suggests that the anions bind to a ‘low-field strength’ positive charge or weak dipole while passing through the channel (Eisenmann 1962; Eisenman & Horn 1983). The channel was also permeant to several other monovalent anions, such as (in increasing order) aspartate, propionate, acetate, Cl^–^, SCN^–^, benzoate and nitrate, with an overall permeability sequence congruent with the lyotropic series (Eisenmann 1962). Prominent within this group were the highly permeable amphipathic SCN^–^ and benzoate ions, which suggested the presence of hydrophobic groups at or near the selectivity site. While running these tests with monovalent anions, we sometimes used the divalent anion SO_4_^2–^ as a counterproof. To some surprise, since it seemed that the channel passed almost any anion, we found that SO_4_^2–^ could not pass (Franciolini & Nonner 1987). This observation proved very useful, as we shall see soon.

#### Cation Selectivity

While analyzing these experiments, we also had a firm impression that cations could also pass through the background Cl^–^ channels. To clear this aspect, we carried out a dedicated set of tests which consisted in probing inside-out patches with varying concentrations of NaCl salines, while keeping the patch electrode at fixed 1,000 mM NaCl solution. With all of the NaCl salines tested, the measured reversal potentials were significantly lower than expected for an ideal Cl^–^ electrode, thus confirming our initial impression (Fig. 7B). Accurate analysis of these results showed that the reversal potentials matched closely the prediction of the Goldman-Hodgkin-Katz voltage equation for the case of *P*_*Na*_*/P*_*Cl*_ equal to 0.2 (Fig. 7B, inset). Similar tests using KCl instead of NaCl gave essentially the same results, that is *P*_*K*_*/P*_*Cl*_ close to 0.2 (not shown). Note, however, that despite the significant alkali cation permeability, when the background Cl^–^ channel was probed with cations only in the presence of the impermeant anion SO_4_^2−^, no cationic current could be observed, as if cations could pass through the channel only if ‘accompanied’ by a permeant anion.

**Fig. 8:**
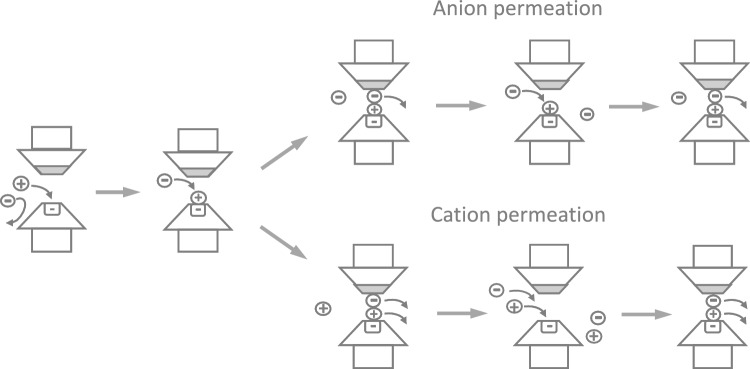
Proposed mechanism to explain the anion and cation passage through the background Cl^–^ channel. See the text for description. [From unpublished drawings sketched by Franciolini and Nonner]

These apparently paradoxical observations were explained by a new mechanism of permeation, which postulates that the background Cl^–^ channel displays, as a key feature, a negative binding site in the selectivity filter, like a cation channel. This implies that the first step is the binding of a permeating cation and the formation of a low-field dipole suitable for attracting a permeating anion (Fig. 8). The binding of an anion to the low-field-strength site would lead to the formation of an activated complex made of the negative binding site, the extrinsic cation, and the extrinsic anion — an activated complex inherently unstable and bound to rapidly decay (Fig. 8). Now, depending on how the activated complex decays, we get either an exclusive anion current or a mixed anion/cation current.

Because energetically favored, most of the time it is the permeant anion that dissociates from the complex and passes through alone, leaving the cation still bound to the channel site and ready to accept a second anion that most likely follows the same fate as the previous anion, thus producing an anionic current (Fig. 8, upper panels). Sometimes, however, the anion and the cation dissociate together from the negative site, and this is how the cation passage takes place (Fig. 8, lower panels). This mechanism does not account for the passage of the cation alone because, due to its strong interaction with the negative site, it is very unlikely that it dissociates from the site before an anion binds to it. Instead, it would do so when bound to a cation because this would weaken its interaction with the negative site. Given the very low probability of a cation crossing the pore on its own, this eventuality was not depicted in the cartoon in Fig. 8. The selectivity structure of the channel was drawn to also contain a hydrophobic region, which is necessary to stabilize and facilitate the passage of amphipathic ions such as SCN^–^ and benzoate, which we have seen to be highly permeant through these channels. A similar mechanism, in which cations and anions can pass through the pore of the antibiotic amphotericin B only as ion pairs, was proposed at about the same time by (Borisova et al. 1986).

Single-channel recording has helped to address many gating and permeation issues of ion channels in many other ways, particularly in partnership with channel structuralists. This has been especially beneficial following advances in crystallography and cryogenic electron microscopy (cryo-EM), which have produced a large number of high-resolution ion channel structures. The single-channel recording approach has been crucial to this partnership because structural information alone is of limited use for understanding how these proteins work unless it is correlated with functional studies.

## Conclusions

The single-channel recording technique, with its many configurations for different experimental needs, has been invaluable in many areas of research over the years. The technique certainly has its limitations, such as low data throughput or the requirement for highly skilled operators. However, there is a general consensus that classical single-channel recording with patch clamp, when performed by expert scientists, remains the technique capable of providing the highest-quality data and the most reliable answers to subtle questions about ion channels. Despite being the oldest single-molecule technique and having changed little over the last 45 years, this technique remains a milestone in the study of protein conformational dynamics. Due to its unparalleled recording length (with many thousands of openings and closures) and sub-millisecond time resolution, it is the only technique that allows one to observe, in real time, when channels open or close (i.e., switch conformation) while remaining in their natural environment within the cell membrane.

## Data Availability

No datasets were generated or analyzed during the current study.
